# Ligand-free gold nanoclusters confined in mesoporous silica nanoparticles for styrene epoxidation†

**DOI:** 10.1039/c9na00781d

**Published:** 2020-03-18

**Authors:** Buthainah Al-Shankiti, Walid Al-Maksoud, Madathumpady Abubaker Habeeb Muhammed, Dalaver H. Anjum, Basem Moosa, Jean-Marie Basset, Niveen M. Khashab

**Affiliations:** Smart Hybrid Materials Laboratory (SHMs), Advanced Membranes and Porous Materials Center, King Abdullah University of Science and Technology (KAUST) Thuwal 23955-6900 Saudi Arabia niveen.khashab@kaust.edu.sa; Division of Physical Sciences and Engineering, KAUST Catalysis Center (KCC), King Abdullah University of Science and Technology (KAUST) 4700 KAUST Thuwal 23955-6900 Saudi Arabia; Advanced Nanofabrication Imaging and Characterization Core Lab, King Abdullah University of Science and Technology (KAUST) Thuwal 23955-6900 Saudi Arabia

## Abstract

We present a novel approach to produce gold nanoclusters (Au NCs) in the pores of mesoporous silica nanoparticles (MSNs) by sequential and controlled addition of metal ions and reducing agents. This impregnation technique was followed to confine Au NCs inside the pores of MSNs without adding external ligands or stabilizing agents. TEM images show a uniform distribution of monodisperse NCs with an average size of 1.37 ± 0.4 nm. Since the NCs are grown *in situ* in MSN pores, additional support and high temperature calcination are not required to use them as catalysts. The use of Au NC/MSNs as a catalyst for the epoxidation of styrene in the presence of *tert*-butyl hydroperoxide (TBHP) as a terminal oxidant resulted in an 88% conversion of styrene in 12 h with a 74% selectivity towards styrene epoxide. Our observations suggest that this remarkable catalytic performance is due to the small size of Au NCs and the strong interaction between gold and the MSNs. This catalytic conversion is environmentally friendly as it is solvent free. We believe our synthetic approach can be extended to other metal NCs offering a wide range of applications.

## Abstract 2

We present a novel approach to produce gold nanoclusters (Au NCs) in the pores of mesoporous silica nanoparticles (MSNs) by sequential and controlled addition of metal ions and reducing agents.

## Graphical abstract

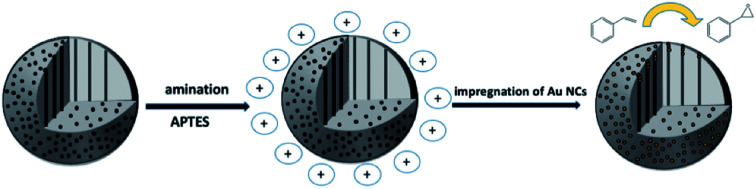

Metal nanoclusters (NCs) are relatively a new class of nanomaterials consisting of only tens of core atoms and display well defined atomic and electronic structures. They are employed in several potential applications such as imaging, sensing, catalysis and solar energy harvesting.^1–6^ NCs with a core size <2 nm display superior catalytic performance compared to plasmonic nanoparticle (NP) and bulk metals due to quantum confinement.^7–11^ Unlike plasmonic NPs, NCs can be prepared with atomic precision and hence it is possible to correlate the structural properties of NCs with their catalytic properties as well as to determine the active sites for catalytic activity. NCs are used in several catalytic reactions including oxidation,^12,13^ hydrogenation,^14,15^ electrocatalysis^16^ and photocatalysis.^17,18^ There are several factors affecting the catalytic properties of NCs such as the nature of capping ligands, doping with other metal atoms, and the morphology and nature of the support.^1^ Unlike their NP counterparts even a minor change in the atomic composition and core size can alter the catalytic properties dramatically. For example, when a single Pd atom was used as a dopant with Au_25_ NCs, the catalytic activity improved significantly either due to the creation of highly active reaction sites on the surface of the NCs or due to the activation of Au sites *via* electronic structure modification.^19^ Therefore it is important to retain the integrity of NCs during catalysis.

For heterogeneous catalysis, NCs are often loaded on inert surfaces such as TiO_2_,^20^ SiO_2_,^21^ carbon nanotubes,^22^*etc.* The presence of a support enhances the robustness, prevents sintering during catalysis and provides excellent recyclability of the NC based catalysts. To attain maximum catalytic activity for NCs, it is important to have an exposed metallic surface to interact with the reactants. Generally, a protecting ligand is used to stabilize and control the size of the metal NCs.^23^ However, the presence of the organic ligand on the surface of the metal NCs may block or reduce the catalytic property in some cases.^24–27^ To avoid this, a ligand that allows the maximum exposed surface area for NC synthesis is used or the ligand is removed from the synthesized NC surface before catalytic application. Several harsh techniques such as CO stripping,^25^ thermal treatment at high temperatures,^28,29^ plasma and UV-ozone cleaning,^30–32^ are employed to remove the coordinated ligands. Though such ligand-off catalysts show higher activity, ligand removal may result in the structure and size modification of NCs and, in some cases, aggregation. Another possibility is to synthesize ligand free NCs *in situ* on proper supports for improved catalysis especially for reactions like styrene epoxidation, which has gained much attention as it is used as a starting material for small molecule drugs and a stabilizer for high molecular weight polymers, and it also plays a major role in the perfume and sweetening industries.^33,34^

Herein, we developed a novel NC based catalyst based on ligand-free Au NCs on mesoporous silica nanoparticle (MSN) supports. The gold precursors were impregnated in the pores of MSNs followed by reduction to obtain NCs. This catalyst was employed in the solvent-free oxidation of styrene using TBHP as an oxidizing agent. Interestingly, the catalyst was utilized without calcination, which was always required in previously reported catalysts.^35–37^

Au NCs were successfully synthesized in the pores of MSNs *via* wet chemical reduction of impregnated gold precursors. For this, amination of MSNs was carried out before introducing an Au precursor to facilitate electrostatic interaction between the MSNs and the added gold precursor ions. This may eliminate the possibility of the formation of NCs in solution outside the silica NPs as well as the aggregation of the formed particles. Furthermore, to control the amination process with (3-aminopropyl)triethoxysilane (APTES), the number of hydroxyl groups on the surface of silica was estimated by titration with methyllithium (MeLi) (quantitative evolution of methane) and found to be 0.4 ± 0.2 mmol of Si–OH per 1 g of MSNs. After the amination process, MSN-NH_2_ was characterized by elemental analysis (CHN) and solid-state NMR spectroscopy. The elemental analysis of MSN-NH_2_, indicated the presence of 2.0 wt% of N (∼1.4 mmol of N grafted on 1 g of MSNs). The atomic ratio of N/OH was calculated to be 3.5, which means that each unit of APTES reacts with 3 units of hydroxyl groups on silica. The C analysis was found to be 10 wt% (∼8.5 mmol of C). The ratio of C/N was calculated to be 5 (expected 3C for N). The excess of carbon content is due to the presence of a solvent (toluene) adsorbed on the support, which was not completely removed after the washing process. Furthermore, MSN-NH_2_ was analyzed by multinuclear solid-state NMR spectroscopy to provide detailed information on the molecular structure of the grafted (≡Si–CH_2_–CH_2_–CH_2_–NH_2_) fragment. The ^1^H NMR spectrum exhibited a significant signal at 2.8, 1.3 and 0.36 ppm arising from protons of Si–C**H**_2_–C**H**_2_–C**H**_2_–N**H**_2_, respectively. The sharp peak at 1.3 ppm is assigned to the protons of NH_2_.

The ^13^C NMR spectrum shows intense peaks at 11.6, 23.0 and 44.5 ppm assigned to the Si–**C**H_2_–**C**H_2_–**C**H_2_–NH_2_ fragment.^38^ Furthermore, two extra peaks appeared at 23 ppm and 129 ppm corresponding to an excess amount of toluene, which was not removed completely during the drying process (Fig. S1†).

TEM images of the MSNs before and after amination are shown in Fig. S3† confirming no change in the size and morphology of MSNs after amination. The size of the MSNs is in the range of 100–150 nm. In the absence of amination, aggregated gold nanoparticles were formed on the surface of MSNs as shown in Fig. S4.† After amination, the zeta potential of the MSNs changed from zero mV to 23.9 mV as shown in Fig. S5.† The formation of Au NCs was initiated by the addition of the Au precursor to the aminated MSNs followed by reduction with NaBH_4_. The dispersion colour changed from light yellow to dark brown after reduction suggesting the formation of NCs. TEM images presented in Fig. 1a showed that MSNs retained their spherical morphology after NC growth. Highly uniform Au NCs with an average size distribution of 1.37 ± 0.4 nm are embedded throughout the MSNs as shown in Fig. 1b. The larger AuNCs of nearly 2 nm size with less population are grown outside the pores of the MSNs and thus there were no constraints on their growth. A 2D projection image of the MSNs (Fig. 1c) confirm the presence of AuNCs inside MSN pores up to a depth of 30 nm. The absence of particle aggregation and the formation of NPs or NCs outside the MSNs proved that NCs can be successfully synthesized inside MSNs *via* our method. There was no aggregation of formed gold nanoclusters as confirmed by high-angle annular dark-field scanning TEM (HAADF-STEM) in Fig. 1d. The elemental composition of AuNCs/MSN-NH_2_ was determined *via* EDX spectroscopy as well elemental mapping (Fig. 1).

**Fig. 1:**
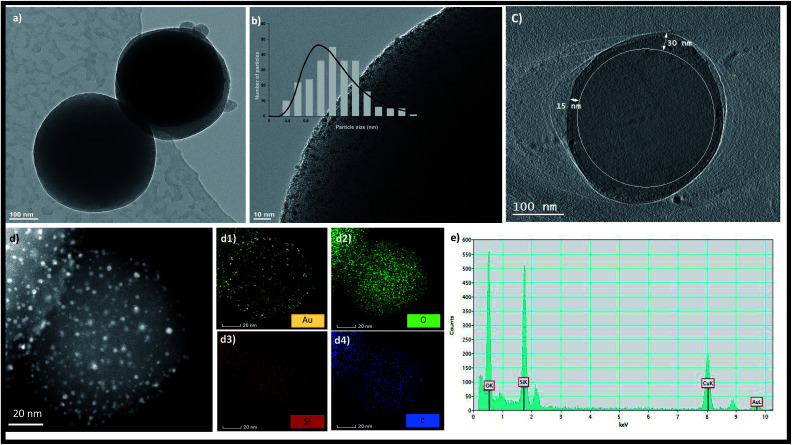
(a and b) Bright-field TEM image, (c) volume-slice electron tomography image. (d) HAADF-STEM image, and STEM EDX mappings of AuNCs/MSN-NH_2_: Au, O, Si, and O are shown in (d1–d4), respectively. (e) EDX spectrum of AuNCs/MSN-NH_2_.

Diffuse reflectance UV-vis spectra of AuNCs/MSN-NH_2_ were measured in the range of 300–800 nm and are shown in Fig. S6.† The absence of the plasmonic peak characteristic of Au nanoparticles (NPs), around 550 nm, confirms that no NPs are formed.

The X-ray photoelectron spectrum (XPS) survey spectrum showed the presence of the elements Au, N, C, O and Si (see Fig. S7†). The atomic percentages of the observed elements are exhibited in the inset of Fig. S7.† The binding energies of gold 4f_7/2_ and 4f_5/2_ shown in Fig. S8† were determined *via* high resolution to be 83.4 and 87.0 eV which are slightly lower than those of the thiolated NCs, which was matching the literature data.^39^ This is expected as the NCs are ligand free (excluding the grafting of amine). From the full range XPS spectrum, sodium and oxygen appear at higher binding energies 1072 eV (Na 1s) and 532 eV (O 1s) as shown in Fig. S7 (ESI†). The remaining peaks at 400 eV, 285 eV and 103 eV correspond to N 1s, C 1s, Si 2p, respectively.

The powder X-ray diffraction data (Fig. S9†) confirm the presence of AuNCs in MSN-NH_2_. Several distinct 2 theta peaks were observed for AuNCs, which show face-centered cubic phases at 39°, 44°, 65° and 79° corresponding to (111), (200), (220) and (311) lattice planes, respectively. The low-angle XRD pattern of MSNs before and after the formation of Au NCs shows an intense peak at 2theta = 1.3°, confirming the ordered mesoporous structure of silica (Fig. S10†) and is in accordance with the literature.^40^ The BET surface area of AuNCs/MSN-NH_2_ is 361 m^2^ g^−1^, which is two times less than the surface area of aminated MSNs (721 m^2^ g^−1^) (ESI†). This further confirms the loading of NCs in the pores of MSNs. The amount of gold loading was defined *via* ICP-OES analysis, to be 10 wt% for high loading and 1 wt% for low loading of gold.

## Catalytic activity of gold clusters

**Scheme 1:**
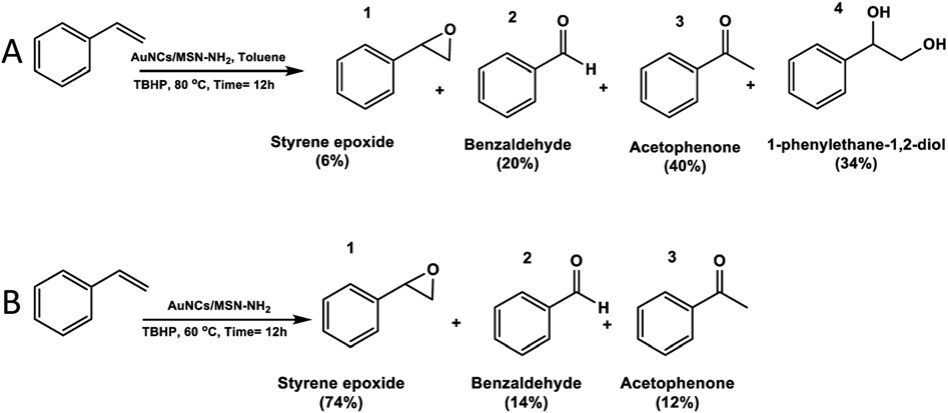
Evidence of the solvent effect in selective styrene epoxidation by using AuNCs/MSN-NH_2_ as a catalyst using a toluene solvent (A) and without a solvent (B).

The selective epoxidation of styrene was investigated to evaluate the catalytic performance of AuNCs/MSN-NH_2_ as shown in (Scheme 1) in the presence of TBHP as an oxidant due to its high performance for alkene epoxidation reactions.^41^

Furthermore, the investigation of the reaction parameters, such as the temperature and the presence of a solvent, has been conducted. During the reaction, various products have been obtained including styrene epoxide (1), benzaldehyde (2), acetophenone (3), and 1-phenylethane-1,2-diol (4) (Scheme 1A and B), which are valuable and extensively used in industry.^42^

The preliminary results of using AuNCs/MSN-NH_2_ as a catalyst for the epoxidation reaction of styrene are shown in Table 1. Using gold clusters Au NCs/MSN-NH_2_ as a catalyst in toluene, a moderate conversion of styrene was obtained (*ca.* 72%) after 12 h at 60 °C.

**Table 1:** The catalytic performance of Au NC/MSN-NH_2_ catalysts for styrene epoxidation with TBHP and in the presence of toluene as a solvent

Catalysts	Temperature (°C)	Time (h)	Conversion (%)	Selectivity for (1)	Selectivity for (2)	Selectivity for (3)	Selectivity for (4)
AuNCs/MSN-NH_2_	60	6	63	24	8	27	41
		12	72	18.5	8	26	47
AuNCs/MSN-NH_2_	80	6	70	20	6	32	42
		12	94	6	20	40	34
		24	100	21.5	4	44	30.5

However, within a similar duration, the catalyst shows an enhanced activity when the reaction temperature was raised to 80 °C. The conversion of styrene reaches up to 95% after 12 h of the reaction. In both cases, the acetophenone (3) and 1-phenylethane-1,2-diol (4) were formed as major products, while styrene epoxide was the minor product (Table 1). The low selectivity of styrene epoxide (1) was attributed to the presence of the solvent, which obstructs the transformation of the electrons from and to gold nanocluster, as it has been observed in previous studies.^43–45^

As reported in the literature, the nature of the solvent plays a major role in the catalytic activity of the catalysts such as conversion and selectivity towards the oxidation reaction of alkenes.^43,46,47^ Moreover, in order to optimize the reaction conditions, the oxidation of styrene was investigated with TBHP in the absence of a solvent at 60 °C (Table 2). Interestingly, a significant influence on the selectivity for styrene epoxide has been observed. The conversion of styrene has reached 88% after 12 h, with a very high selectivity for styrene epoxide (*ca.*74%). It is worth noting that under the same conditions, pristine MSN-NH_2_ shows a very low conversion of styrene (less than 17%).

**Table 2:** The catalytic performance of AuNCs/MSN-NH_2_ and pristine MSN-NH_2_ catalysts for styrene epoxidation with TBHP (solvent-free) at 60 °C

Catalysts	Time (h)	Conversion (%)	Selectivity for (1)	Selectivity for (2)	Selectivity for (3)
AuNCs/MSN-NH_2_	3	55	73	18	9
	6	83	78	15	8
	12	88	74	14	12
Pristine MSN-NH_2_	3	6	75	4	21
	6	11	76	5	19
	12	17	74	7	19

A kinetic study was performed under the optimized conditions (solvent-free) at different temperatures (60 °C and 80 °C) (Fig. S17†) for 6 h. In addition, a blank test was performed at 60 °C in the presence of pristine MSN-NH_2_. The obtained results show that the catalytic activity of AuNCs/MSN-NH_2_ at 80 °C was higher compared to that at 60 °C at the beginning of the reaction; without any appearance of the induction period. The initial activity of AuNCs/MSN-NH_2_ at 80 °C is 10.9 mmol g^−1^ [Au] h^−1^, whereas the activity was 6.4 mmol g^−1^ [Au] h^−1^ at 60 °C. However, pristine MSN-NH_2_ shows a very low initial activity with an induction period of 60 min (curve red). As for the selectivity for styrene epoxide (1), a constant selectivity (75 ± 5%) has been obtained during the reaction under either a temperature of 80 °C or 60 °C (Fig. S17†).

**Fig. 2:**
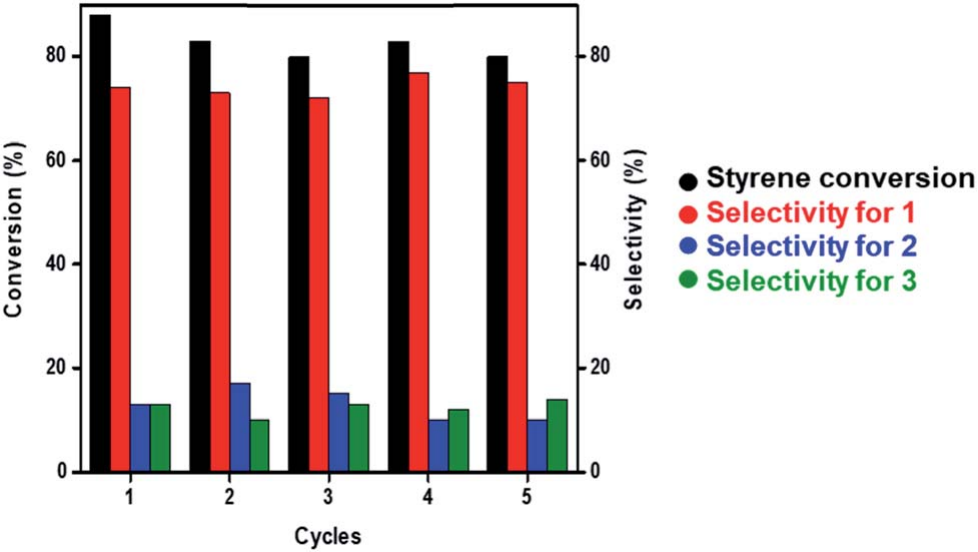
Recycling graph of styrene epoxidation at 60 °C using the AuNC/MSN-NH_2_ catalyst.

Under optimized reaction conditions (60 °C, over 12 h, and solvent-free), we attempted to study the recyclability of Au NCs/MSN-NH_2_ during the epoxidation reaction of styrene. After the first run of the catalyst, the reaction mixture was allowed to cool to room temperature, and the catalyst was separated by centrifugation, washed twice with ethanol and allowed to dry at room temperature. The recycled gold catalyst was then used without any regeneration under the same reaction conditions. The conversion of styrene dropped from 88% to 80% after 5 cycles (Fig. 2). The selectivity of styrene epoxide (1) was consistent (∼75%) during the 5 cycles. Furthermore, the weight percent of gold leaching after 5 runs was examined by ICP-OES to be 3.6 wt% out of 10 wt%.

**Scheme 2:**
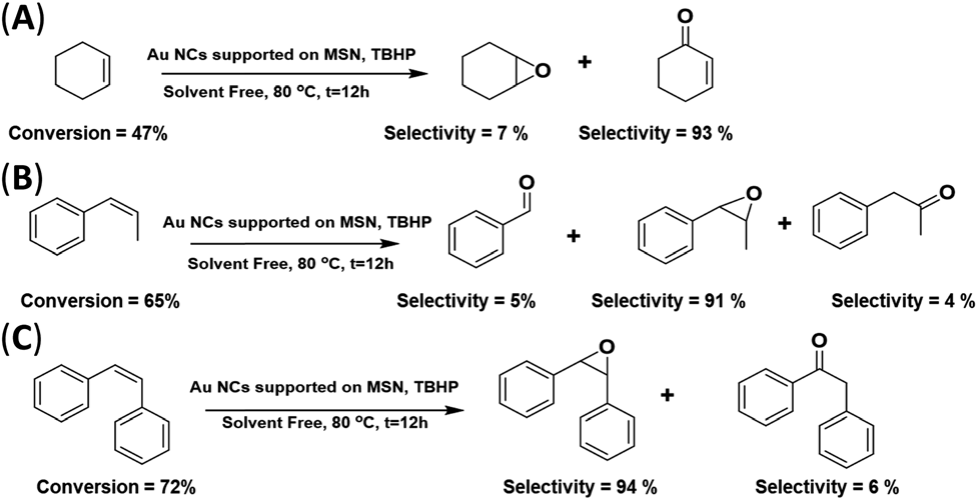
Epoxidation of various substrates (cyclohexene, 1-phenylpropene and *cis*-stilbene) using the AuNC/MSN-NH_2_ catalyst (solvent free).

The catalytic activity of the AuNC/MSN-NH_2_ catalyst was further tested with various olefins; the reaction was carried out under the above-mentioned conditions. As clarified in Scheme 2(A), the conversion of cyclohexene is 47% after 12 h at 80 °C, when the C=C is oxidized and two products are formed. Cyclohexene epoxide (∼7% selectivity) and cyclohexen-1-one (∼93% selectivity). The dominance of cyclohexene-1-one over cyclohexene oxide was comparable to the best reported results of cyclohexene epoxidation in the presence of Au/C.^43,48^ Interestingly, only titanium silicate (TS-1) gives 100% selectivity for cyclohexene epoxide.^49^ This low selectivity for cyclohexene oxide may be restricted to non-aromatic alkenes with this catalyst. The double bond in cyclohexene interacts differently with the AuNC surface in comparison with aromatic alkenes. The disappearance of other side products was attributed to the absence of a solvent. On the other hand, a moderate conversion of phenylpropene was achieved (∼65%), and the selectivity for phenylpropene oxide was very high (91%) (Scheme 2(B)). In addition, it was found that stilbene oxide was produced with a high selectivity of 95% at a conversion of 72% of *cis*-stilbene (Scheme 2(C)). It is worth noting that previous studies have reported high efficiency and selectivity for olefin epoxidation in the absence of any solvent.^50^ The high conversion of *cis*-stilbene and 1-phenylpropene compared to cyclohexene is attributed to the stability and the position of the double bond. The internal alkene double bonds are more stable and less accessible by the catalyst compared to the external double bonds.^51^ Therefore, the external π-electrons in *cis*-stilbene and 1-phenylpropene are favourable for the chemisorption of the aromatic rings onto the gold surface and thus, the approach of the double bond to the surface. In cyclohexene, however, such an approach is not favourable.

In conclusion, ligand-free Au NCs confined in MSN-NH_2_s were synthetized successfully by using the chemical impregnation technique. Mesoporous silica with 2–3 nm pore size was used to control the size of Au NCs. The average size of Au NCs was estimated with HR-TEM to be 1.37 ± 0.4 nm. The synthesized catalyst was characterized by NMR spectroscopy, zeta potential measurements, BET, ICP-OES analysis, X-ray diffraction (XRD), X-ray photoelectron spectroscopy (XPS) and transmission electron microscopy (TEM) techniques. The catalytic activity of this material was systematically investigated *via* a solvent-free oxidation reaction of styrene. Interestingly, AuNCs/MSN-NH_2_ can achieve an 88% conversion of styrene with a 74% selectivity towards styrene oxide. Our heterogeneous catalyst is sufficiently stable and can be successfully recovered and reused for five runs. Furthermore, the oxidation of cyclohexene, 1-phenylpropene and *cis*-stilbene to the corresponding epoxide was detected with high selectivity. This is a “green” approach to petrochemical epoxidation where the use of organic solvents and high temperatures are not required. We believe that this strategy could be applied to a wide range of noble metals which drastically increases the possibility of selective catalytic reactions.

## Author contributions

B. S. and W. M. contributed equally. The manuscript was written through contributions of all authors.

## Conflicts of interest

The authors declare no conflict of interest.

## Supplementary Material

NA-002-C9NA00781D-s001

NA-002-C9NA00781D-s002
