# Supplementary material for: Ligand-free gold nanoclusters confined in mesoporous silica nanoparticles for styrene epoxidation
Source: Nanoscale Adv. 2020 Mar 18;2(4):1437–42. doi: 10.1039/c9na00781d (PMC9417287; doi:10.1039/c9na00781d)
Supplement: NA-002-C9NA00781D-s001 [file NA-002-C9NA00781D-s001.pdf]

Electronic Supplementary Material (ESI) for Nanoscale Advances.
This journal is © The Royal Society of Chemistry 2020

Supporting information

Ligand- Free Gold Nanoclusters Confined in Mesoporous Silica Nanoparticles for Styrene Epoxidation

Buthainah Alshankiti, [†‡] Walid Almaksoud, [§‡] Madathumpady Abubaker Habeeb Muhammed, [†] Arnaud Chaix, [†] Dalaver H. Anjum, [¥] Basem Moosa, [†] Jean-Marie Basset, [§] Niveen M. Khashab *[†]

[†] Smart Hybrid Materials Laboratory (SHMs), Advanced Membranes and Porous Materials Center, King Abdullah University of Science and Technology (KAUST), Thuwal 23955-6900, Saudi Arabia
[§] Division of Physical Sciences and Engineering, KAUST Catalysis Center (KCC), King Abdullah University of Science and Technology (KAUST), 4700 KAUST, Thuwal 23955-6900, Saudi Arabia
[¥]Advanced Nanofabrication Imaging and Characterization Core Lab, King Abdullah University of Science and Technology (KAUST), Thuwal 23955-6900, Saudi Arabia

EXPERIMENTAL SECTION

Materials. Tetraethyl orthosilicate (TEOS), (3-aminopropyl)triethoxysilane (APTES), cetyltrimethylammonium bromide (CTAB), gold(III) chloride ($HAuCl_4$), sodium borohydride ($NaBH_4$), tert-Butyl hydroperoxide **(**TBHP), Styrene, dichloromethane (DCM), Tetrahydrofurane, cyclohexene, 1-phenylpropene, cis-stilbene, toluene, methyllithium and methanol were purchased from Sigma-Aldrich. Al chemicals were used without further purification.

Instrumentation. Transmission electron microscopy (TEM) of samples was performed by using a microscope of model Titan ThemisZ from Thermo-Fisher Scientific. The specimens of AuNCs loaded onto MSN-NH_2 were prepared first. This has been done by dispersing a small amount of samples each time into ethanol and then drop-casted on carbon-coated Cu grids. The Cu grids containing samples were air dried for several hours prior to TEM experiments. TEM analysis was consisted of imaging of "AuNCs loaded MSN-NH_2 particles" in TEM mode, recording of the crystal structure in selected area electron diffraction (SAED) mode, elemental mapping in scanning TEM (STEM) mode combined with energy dispersive spectroscopy (EDS), and finally 3-dimensional imaging with electron tomography (ET) mode. It should be noted that the imaging of samples in TEM mode allowed determining the particle size and morphology along with the concentration of AuNCs onto MSN particles. Whereas the acquisition of SAED patterns enabled in finding out the crystal structure of AuNCs. Basically, it helped in determining the fact whether the AuNCs were present in face-centered Cubic (FCC) phase or in amorphous phase. Elemental mapping turned out to be useful way of knowing whether the AuNCs were present in the pores of MSN-NH_2 particles. It is noted herein that high-angle annular dark-field (HAADF) detector was chosen to carry out the STEM-EDS mapping experiments. It was done this way because image-contrast in HAADF-STEM mode are highly sensitive to atomic number (Z) of elements and hence it was presumed that the images of Au clusters will become readily visible in this mode. Finally, the ET experiments were carried out to determine the morphology and shape of both MSN-NH_2 particles and AuNCs in 3-dimensions. It is to be noted that the samples were tilted to +/- 70 degrees with an increment interval of 2 degrees during the ET data-acquisitions. The acquired data-sets had been aligned and reconstructed in weighted-back-projected (WBP) schemes available in IMOD software package which is available freely on the Web (https://bio3d.colorado.edu/imod/).[1]
Zeta-potential analyses were performed using a Malvern Nano ZS instrument. Trace-metal analysis carried out using inductively coupled plasma optical emission spectrometry (ICP-OES) on a Varian 720-ES ICP-optical emission spectrometer. Dried powdered samples of Au NCs/ MSN-NH_2 were first acid digested with aqua regia and then diluted prior to measurements. GC measurements were performed with an Agilent 7890A Series (FID detection). We used the following method for GC analyses; column HP-5, 30 m length × 0.32 mm i.d. × 0.25 µm film thickness, flow rate = 1 mL/min (N_2), split ratio = 50/1,

inlet temperature = 250 °C, detector temperature = 250 °C, temperature program = 40 °C/min), 300 °C (3 min). GC−MS measurements were performed with an Agilent 7890A series coupled with Agilent 5975C series. GC/MS equipped with a capillary column coated with nonpolar stationary phase HP-5MS was used for molecular weight determination and identification that allowed the separation of hydrocarbons according to their boiling point differences. The BET surface area, total pore volume and pore size of MSN-NH_2 and MSN-$NH_{2/}$ Au NCs were determined with the Micromeritics ASAP 2420 instrument. X-ray photoelectron spectroscopy (XPS) studies were carried out in a Kratos Axis Ultra DLD spectrometer, equipped with a monochromatic Al Kα X-ray source (hv= 1486.6 eV) operating at 150 W, a multi-channel plate and delay line detector under a vacuum of ~10^{-9} mbar. The survey and high-resolution XPS spectra were analyzed at fixed analyzer pass energies of 160 eV and 20 eV, respectively. The material was dispersed in MeOH then dropcast over silicon substrate. Powder X-ray diffraction was recorded on a Bruker AXS D8 diffractometer using Cu-Kα radiation. One-dimensional 1H MAS and 13C CP/MAS solid state NMR spectra were recorded on Bruker AVANCE III spectrometers operating at 600 MHz resonance frequencies for 1H. Experiments at 600 MHz employed a conventional double-resonance 3.2 mm CP/MAS probe. Dry nitrogen gas was utilized for sample spinning to prevent degradation of the samples. NMR chemical shifts are reported with respect to the external references TMS and adamantane. For 13C CP/MAS NMR experiments, the following sequence was used: 90° pulse on the proton (pulse length 2.4 s), then a cross-polarization step with contact time of typically 2 ms, and finally acquisition of the 13C signal under high-power proton decoupling. The delay between the scans was set to 5 s to allow the complete relaxation of the 1H nuclei, and the number of scans ranged between 10000 and 20000 for 13C and was 32 for 1H. An exponential apodization function corresponding to a line broadening of 80 Hz was applied prior to Fourier transformation.
The 2D 1H−13C heteronuclear correlation (HETCOR) solid state NMR spectroscopy experiments were conducted on a Bruker AVANCE III spectrometer using a 3.2 mm MAS probe. The experiments were performed according to the following scheme: 90° proton pulse, t1 evolution period, CP to 13C, and detection of the 13C magnetization under TPPM decoupling. For the cross-polarization step, a ramped radio frequency (RF) field centered at 75 kHz was applied to the protons, while the 13C channel RF field was matched to obtain an optimal signal. A total of 64 t1 increments with 2000 scans each were collected. The sample spinning frequency was 15 kHz. Using a short contact time (0.2 ms) for the CP step, the polarization transfer in the dipolar correlation experiment was verified to be selective for the first coordination sphere to lead to correlations only between pairs of attached 1H−13C spins (C−H directly bonded).

Synthesis of MSN. Mesoporous silica nanoparticles (MSN) were prepared by dissolving CTAB (250 mg, 0.67 mmol) in deionized water (120 mL) then an aqueous solution of NaOH (875 µL, 2 M) was added. The mixture was stirred for 30 min and heated, when the temperature reached up to 80 °C, TEOS (1.25 mL, 5.6 mmol) was slowly added. The sol-gel process was conducted for 2 h, and the solution was then cooled down to room temperature. Then collected by centrifugation and washed with water and ethanol to remove residual reactants. Subsequently, the product was dissolved in ethanol with 500 µl HCl, then kept under heating at 60°C for 6 hrs. in order to remove the template (CTAB). The product was collected via centrifugation and washed with ethanol and acetone. Then it was kept to dry under atmosphere pressure.
Quantify the amount of hydroxyl group on the surface of silica. The interaction between methyllithium (CH_3Li) and silica considered one of the most reliable method to estimate the number of OH group (the silanol number α_{OH}).[2]
According to the stoichiometry in the equation of the reaction, the amount of the produced methane will be equivalent to the content of surface OH group.

$$\equiv Si-OH + CH_3Li \longrightarrow \equiv Si-OLi + CH_4$$

The concentration of surface hydroxyl group ($\alpha_{OH(S)}$) on SiO_2 surface, referred to the unit mass of the sample (mmol of OH/g of SiO_2). It was determined to be 4 ± 0.2 mmol OH/silica.

Amination of silica nanoparticles (MSN-NH_2). 50 mg of MSN was dissolved in 5 ml dry toluene containing 12.5 µL APTES (3-aminopropyl) triethoxysilane and the solution was left under heating overnight at 120°C. The solution was centrifuged and the precipitate was washed with toluene, THF and methanol, respectively. The precipitate was collected to dry under 60 °C.

The CHN elemental analysis was performed for MSN before and after the amination process.
MSN: 2% of C and $\simeq$ 0 % of N, **MSN-NH_2:** 2.16 % of N, 10.60 % of C and 4.42 % of H.
The low percentage of C in MSN comes from unreacted TEOS, and the high carbon percentage in MSN-NH_2 related to the grafting of APTES on the surface of silica.

According to the silanol number and the elemental analysis we can conclude that each one unit of APTES attached to 3 hydroxyl group on the surface of silica.

Impregnation of Au NCs in the pores of MSN- NH_2. The impregnation technique was followed in order to adjust the amount of the gold precursors needed to grow inside the pores of MSN.[3] So that, the volume of gold precursor solution will be equal to MSN- NH_2 pore volume.
For low loading of gold NCs, an aqueous solution of $HAuCl_4$ (7µL, 100 mM) was added to MSN-NH_2 (60 mg) dispersed in 1 mL of methanol followed by continuous stirring for 3 hours under ambient conditions. Afterwards, the mixture was centrifuged and washed twice with water then with methanol to remove unreacted $HAuCl_4$ and was dried overnight under reduced pressure at room temperature. Subsequently the dried MSN/Au particles was dispersed in 1.5 mL of methanol and cooled to 0°C. This was followed by the addition of methanolic solution of $NaBH_4$ (14 mg, in 1 mL methanol) under vigorous stirring. The reaction was continued for 45 min for the complete reduction of impregnated Au(III) ions to Au(0) in the pores of MSN- NH_2 generating the Au nanoclusters. The color of the reaction mixture changed from light yellow to dark brown and the product so formed was collected by centrifugation and washed with water and methanol several times. In the same way, high loading of Au NCs was prepared but the concentration of gold precursor was ten times more than the previous one (70 µL,100 mM).

Solid-State Nuclear Magnetic Resonance Spectroscopy.

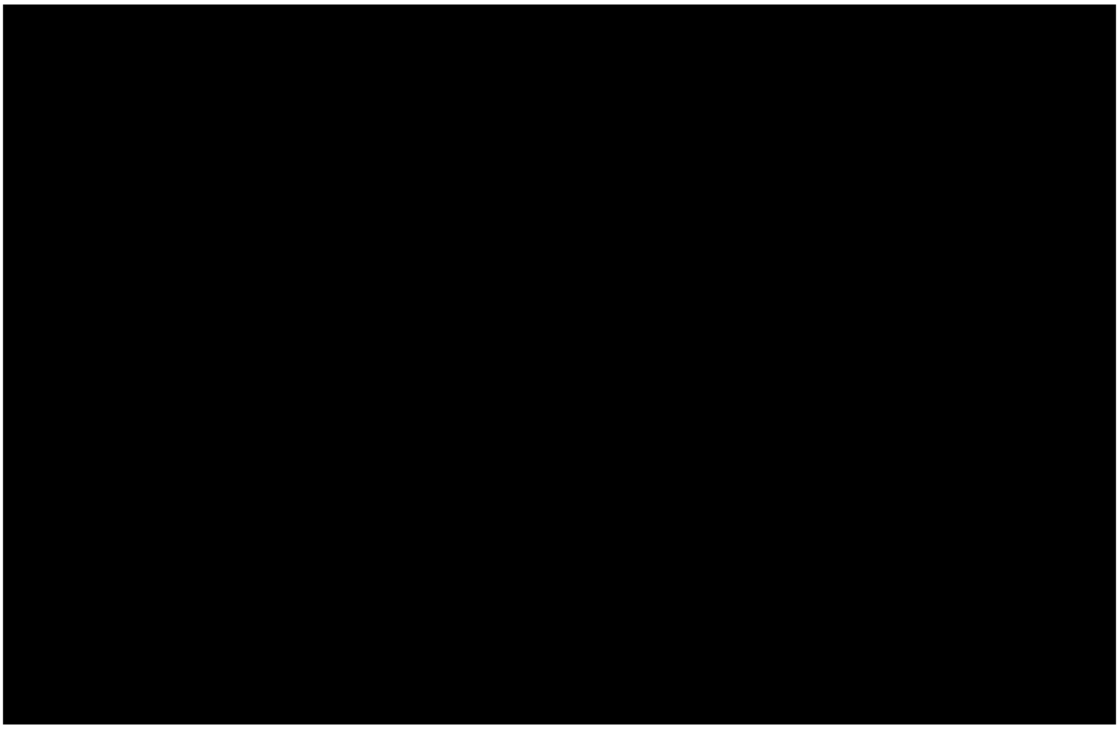

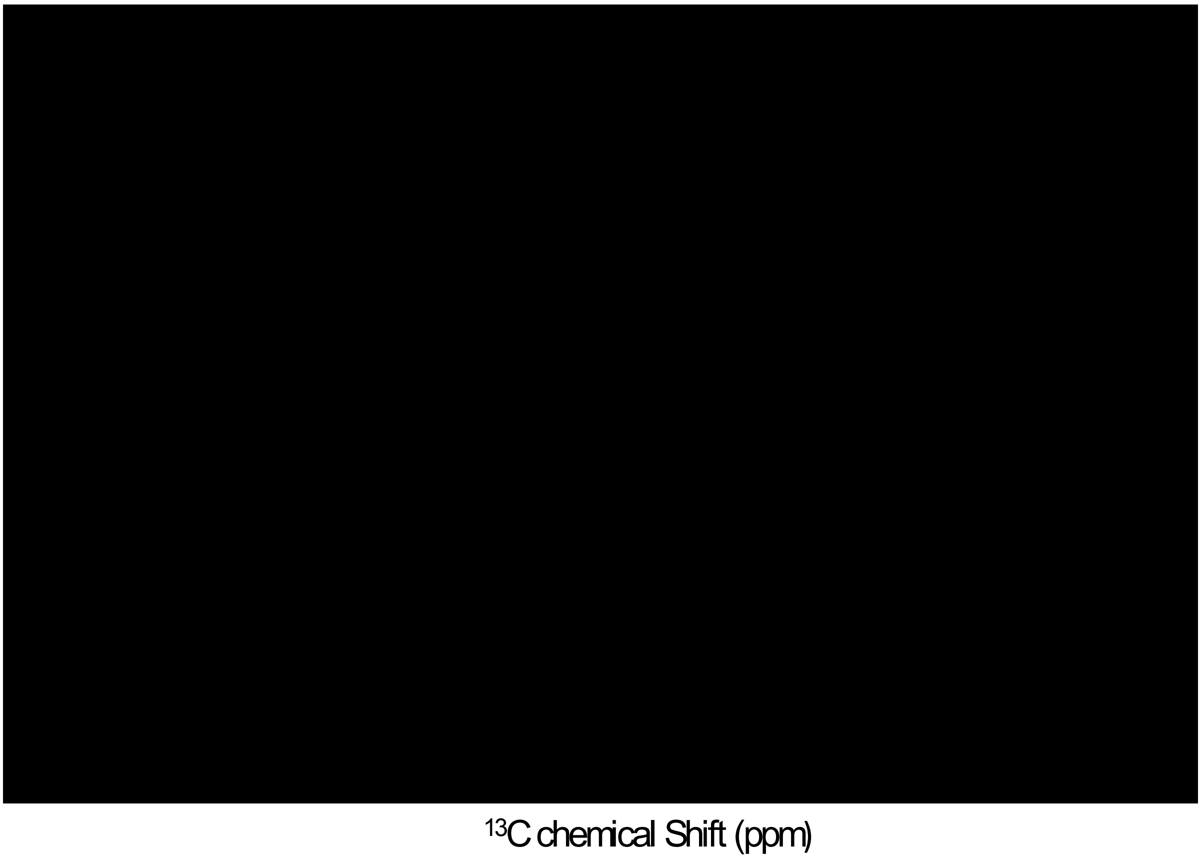

Figure S1: The (a) proton & (b) carbon-13 NMR spectrum of $MSN\text{-}NH_2$.

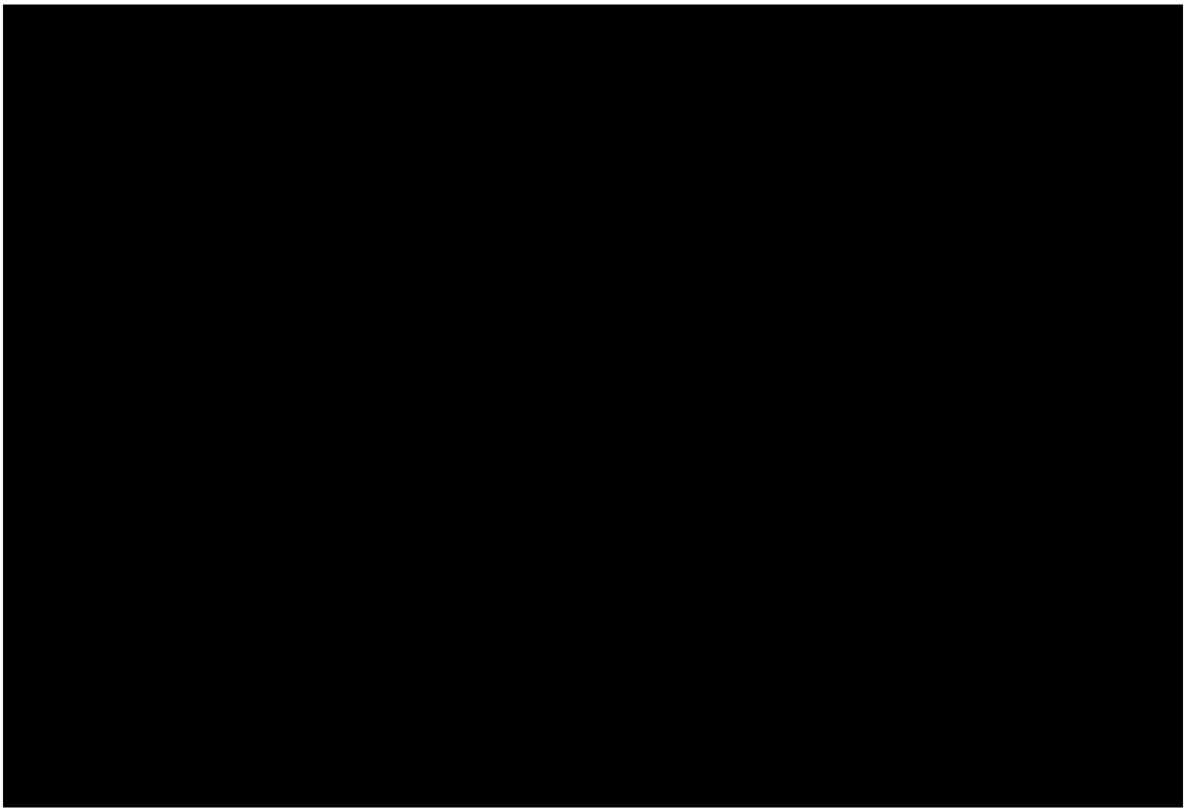

^{1}H chemical Shift (ppm)

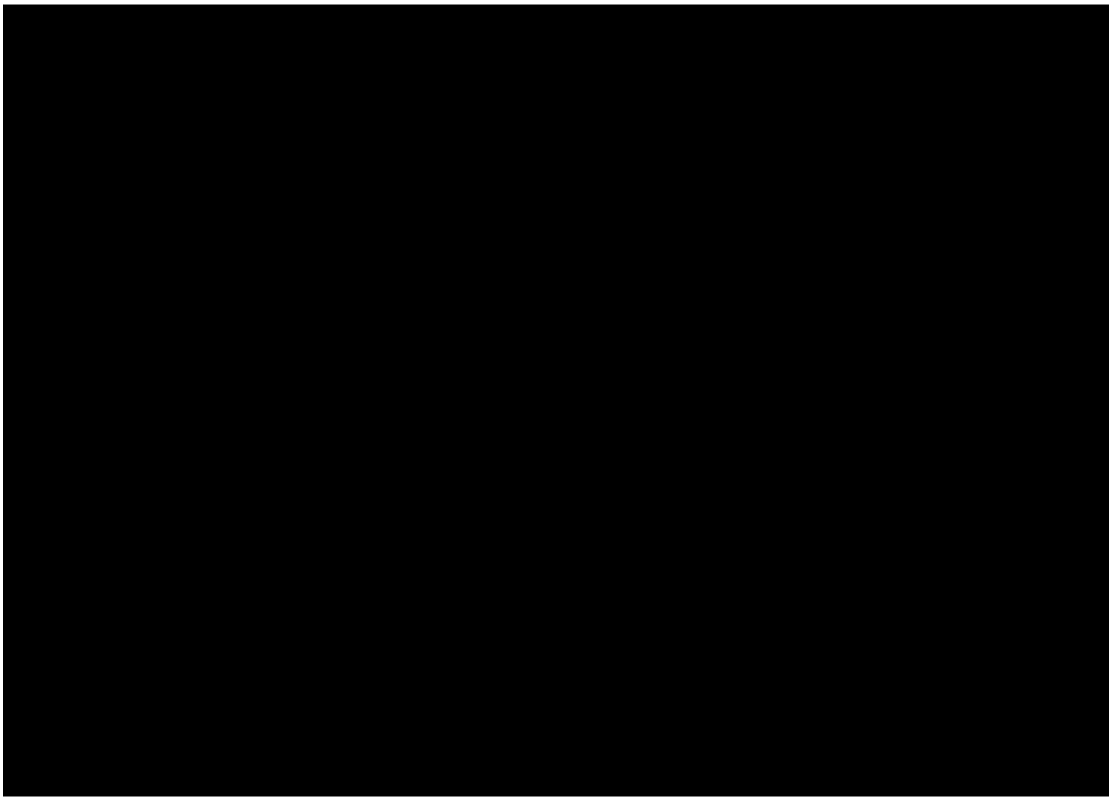

^{13}C chemical Shift (ppm)

Figure S2: The proton (a) & carbon-13 (b) NMR spectrum of AuNCs/ MSN-NH_2.

Catalytic Experiments

1- **Oxidation of styrene**

With solvent: TBHP as an oxidant: $MSN-NH_2$/ gold catalysts (50 mg powder, 10 % wt loading of Au (0.025mmol)) were mixed with styrene (100 µL, 0.87 mmol), toluene (2 mL) and TBHP (0.5 mL, 5.5 mmol) in an ampoule. The ampoule was frozen outside using liquid nitrogen and sealed under vacuum. The sealed reactor was heated under vigorous stirring at 353 K or/and 333 K for 6, 12, and 24h. At the end of the reaction the ampules were taken outside, frozen under liquid nitrogen and then quenched with dichloromethane. The reaction mixture was filtered, the filtrate was collected and was analyzed by GC and GC-MS.

2- **Solvent-free:** TBHP as an oxidant: $MSN-NH_2$/ gold catalysts (50 mg powder, 10 % wt. loading of Au (0.025 mmol)) were mixed with styrene (100 µL, 0.87 mmol)), and TBHP (200 µL, 2.2 mmol) in an ampoule. The ampoule was frozen outside using liquid nitrogen and sealed under vacuum. The sealed reactor was heated under vigorous stirring at 353 K or/and 333 K for 6, 12, and 24h. At the end of the reaction the ampules were taken outside, frozen under liquid nitrogen and then quenched with dichloromethane. The reaction mixture was filtered, the filtrate was collected and was analyzed by GC and GC-MS.

3- **Oxidation of cyclohexene**

TBHP as an oxidant: $MSN-NH_2$/ gold catalysts (50 mg powder, 10 % wt loading of Au, 0.025 mmol) were mixed with cyclohexene (100 µL, 1mmol), and TBHP (200 µL, 2.2 mmol) in an ampoule. The ampoule was frozen outside using liquid nitrogen and sealed under vacuum. The sealed reactor was heated under vigorous stirring at 353 K for 12. At the end of the reaction the ampules were taken outside, frozen under liquid nitrogen and then quenched with dichloromethane. The reaction mixture was filtered, the filtrate was collected and was analyzed by GC and GC-MS.

4- **Oxidation of 1-phenylpropene**

TBHP as an oxidant: $MSN-NH_2$/ gold catalysts (50 mg powder, 10 % wt loading of Au, (0.025mmol)) were mixed with phenylpropene (100 µL, 0.77mmol), and TBHP (200 µL, 2.2 mmol) in an ampoule. The ampoule was frozen outside using liquid nitrogen and sealed under vacuum. The sealed reactor was heated under vigorous stirring at 353 K for 12. At the end of the reaction the ampules were taken outside, frozen under liquid nitrogen and then quenched with dichloromethane. The reaction mixture was filtered, the filtrate was collected and was analyzed by GC and GC-MS.

5- **Oxidation of cis-stilbene**

TBHP as an oxidant: $MSN-NH_2$/ gold catalysts (50 mg powder, 10 % wt loading of Au, (0.025mmol)) were mixed with stilbene (100 µL, 0.55), and TBHP (200 µL, 2.2 mmol) in an ampoule. The ampoule was frozen outside using liquid nitrogen and sealed under vacuum. The sealed reactor was heated under vigorous stirring at 353 K for 12. At the end of the reaction the ampules were taken outside, frozen under liquid nitrogen and then quenched with dichloromethane. The reaction mixture was filtered, the filtrate was collected and was analyzed by GC and GC-MS

6- **Recyclability**

The catalyst stability was explored by its activity and recyclability. The reaction parameters applied for the recyclability test was the same for the one described in styrene epoxidation. Each run was done at 60° C for 12h in the presence of TBHP as oxidizing agent. After the mixture cool down, the solid catalyst was isolated from the reaction mixture. Then it was washed by centrifugation and with ethanol. Later on, it was dried under vacuum and reutilized in the subsequent run

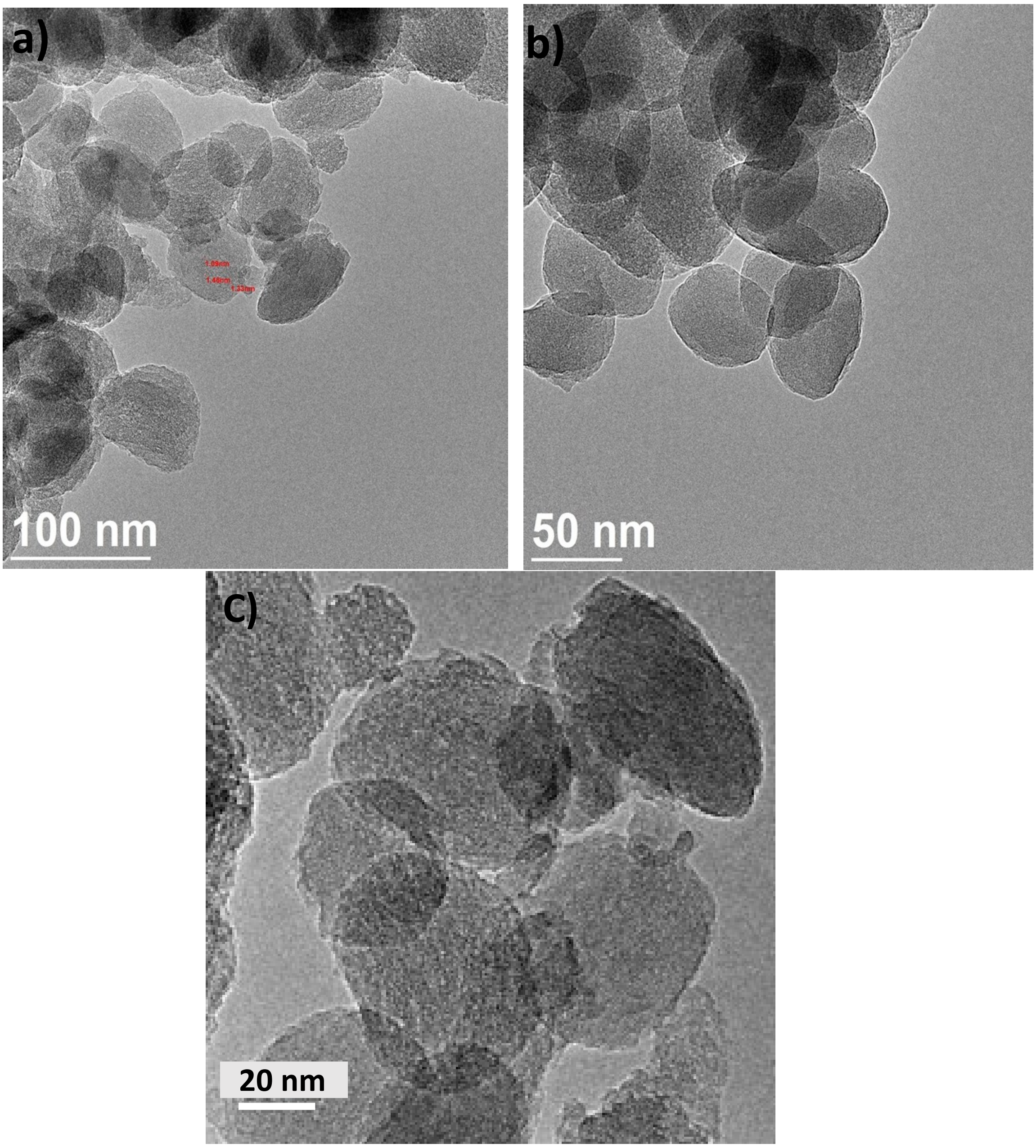

Figure S3. TEM images of MSN NPs before (a) and after amination (b). HR-TEM image of MSN NPs(C).

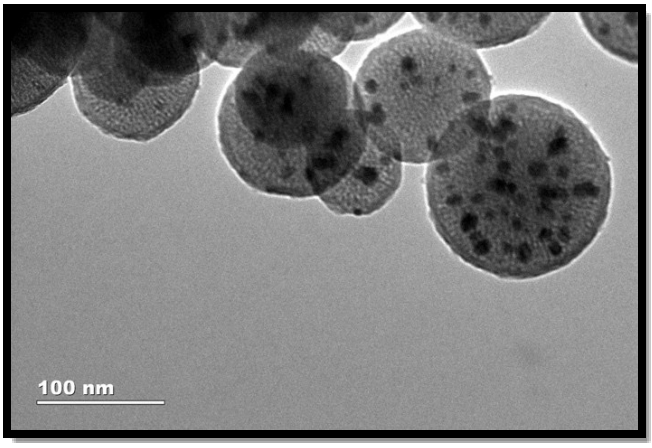

Figure S4.TEM images of aggregated gold nanoparticles on the surface of mesoporous silica nanoparticles.

a)

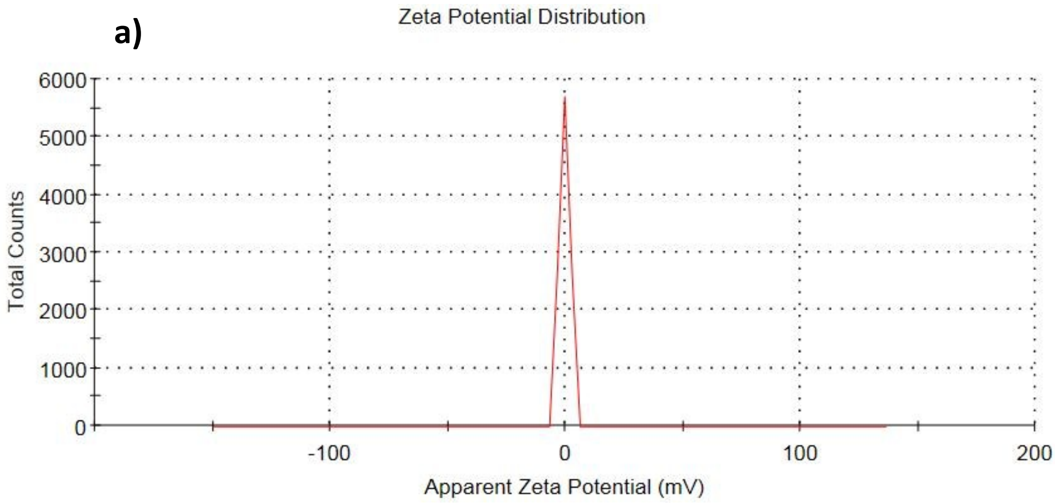

b)

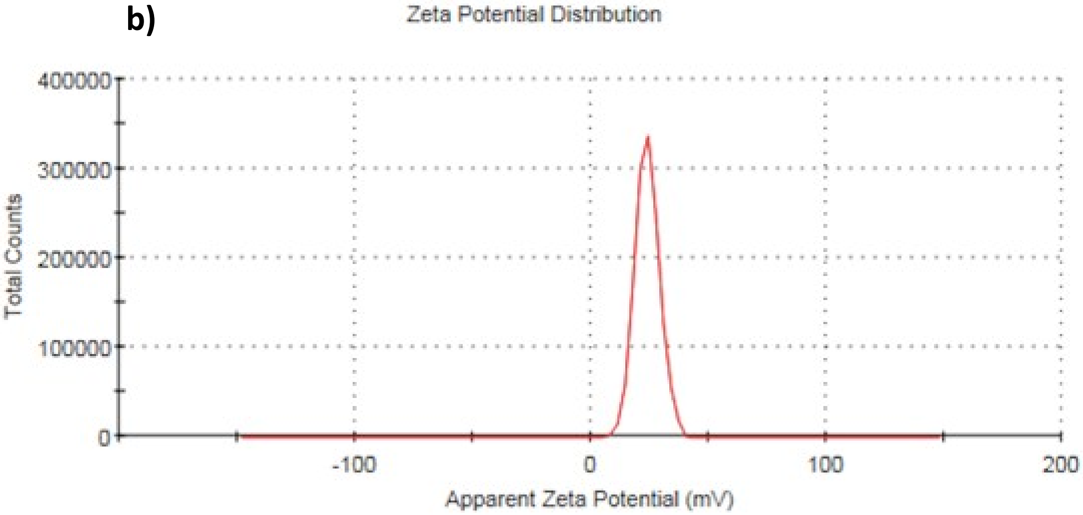

Figure S5. Zeta potential measurements of MSN zero mV (a) NH_2-MSN 23.9 mV (b).

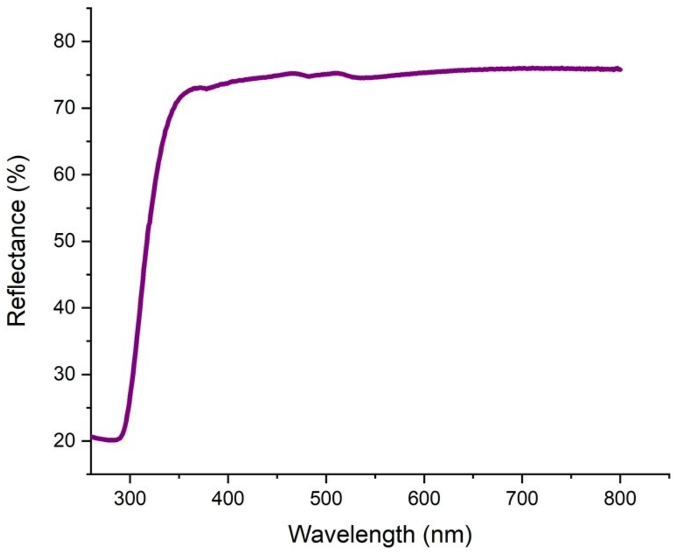

Figure S6. Diffuse reflectance UV-vis spectra of AuNC@MSN.

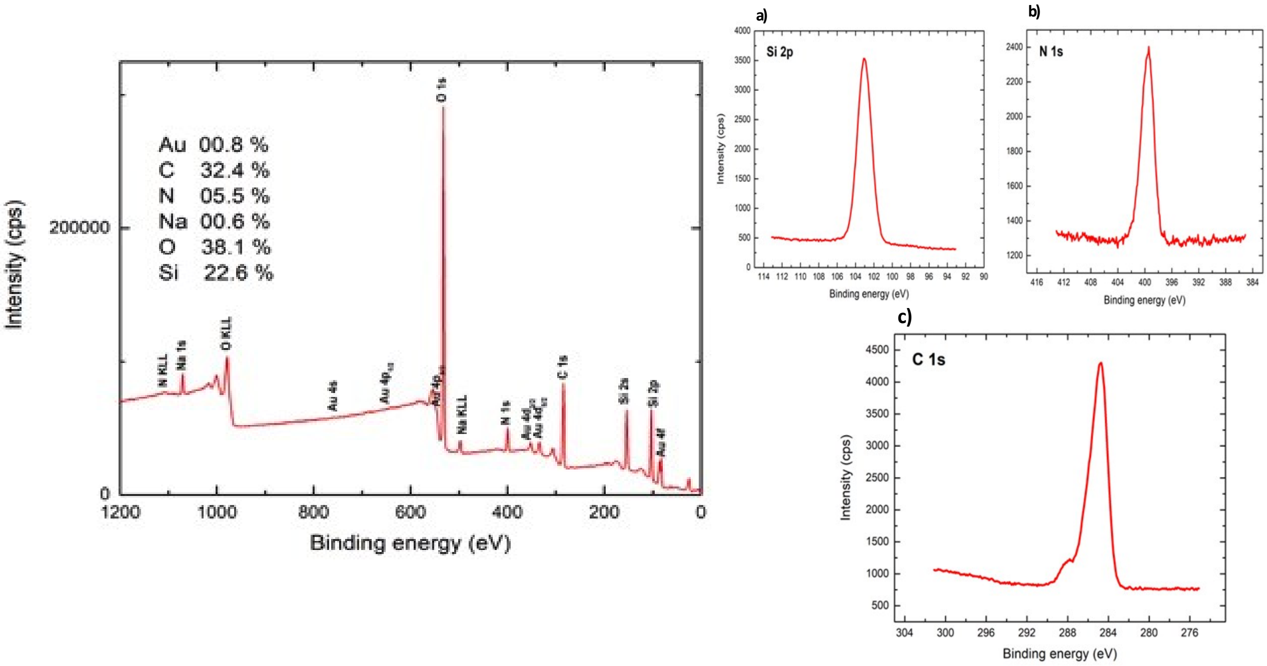

Figure S7. XPS survey spectrum of Au NCs/ NH_2-MSN.

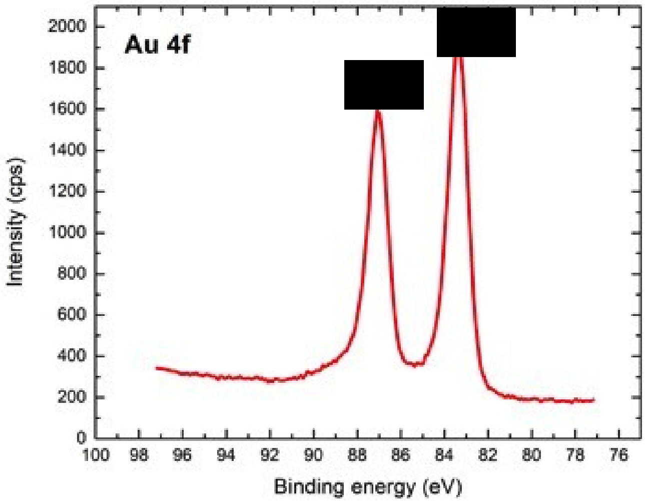

Figure S8: High-resolution XPS Au 4f spectrum of Au NCs/ MSN-NH_2

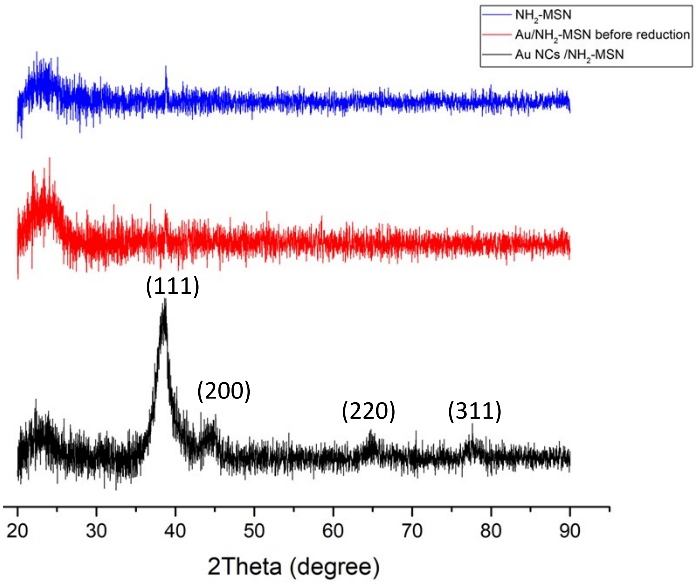

Figure S9. Wide-angle X-ray scattering of MSN-NH_2 and AuNCs/MSN-NH_2 before and after the reduction.

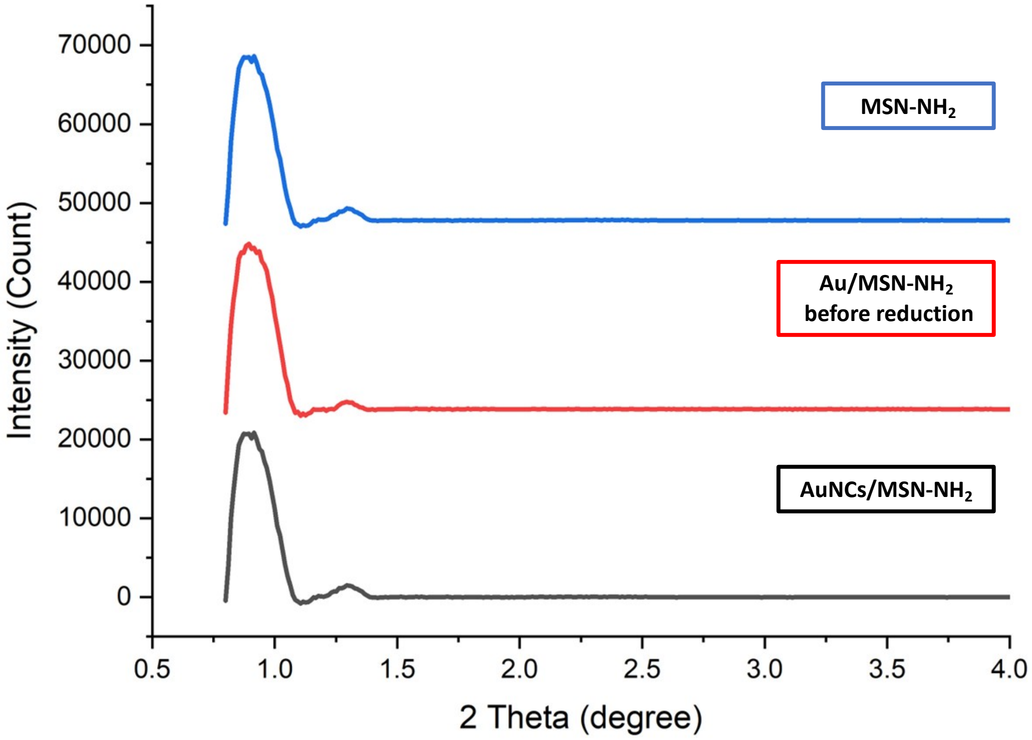

Figure S10. Small-angle X-ray scattering of MSN-NH_2 and AuNCs/MSN-NH_2.

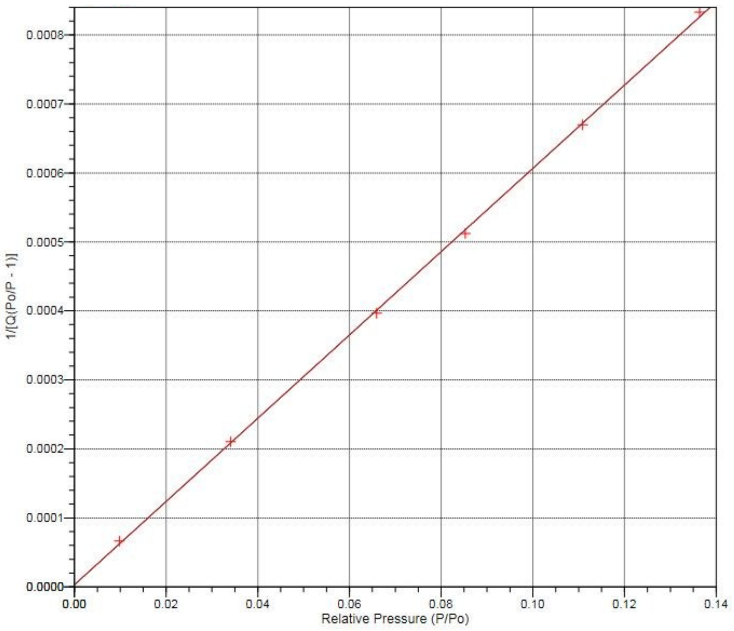

Figure S11. BET surface area of mesoporous silica nanoparticles.

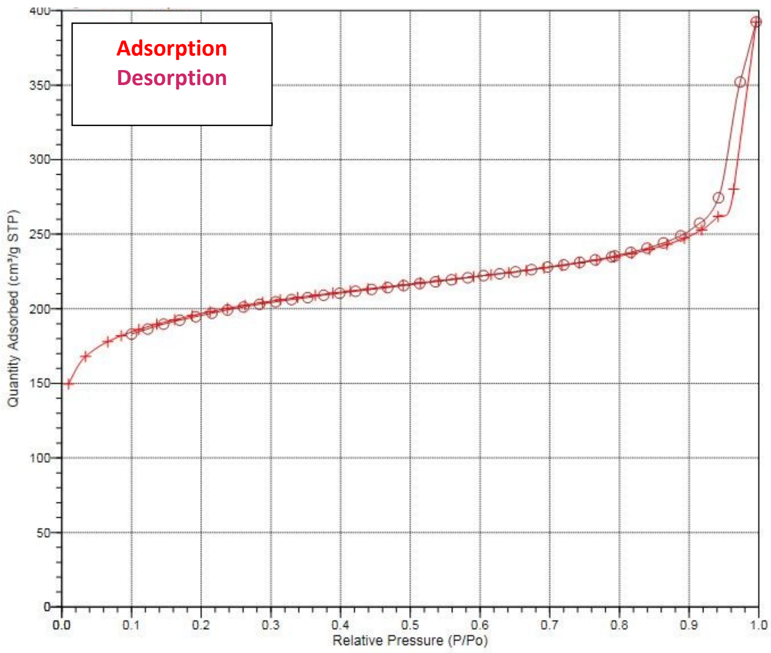

Figure S12. Isotherm linear plot of mesoporous silica nanoparticles, showing type IV isotherm.

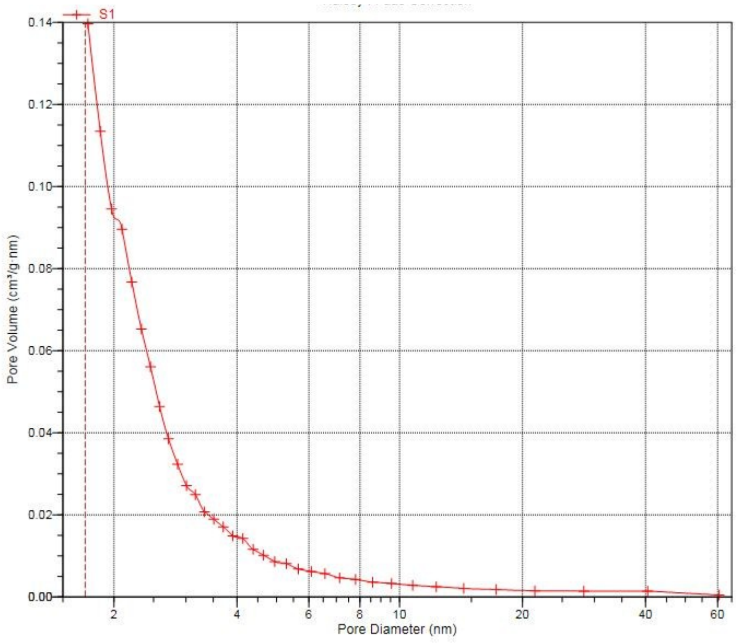

Figure S13. Differential pore volume of mesoporous silica nanoparticles determined using BJH model.

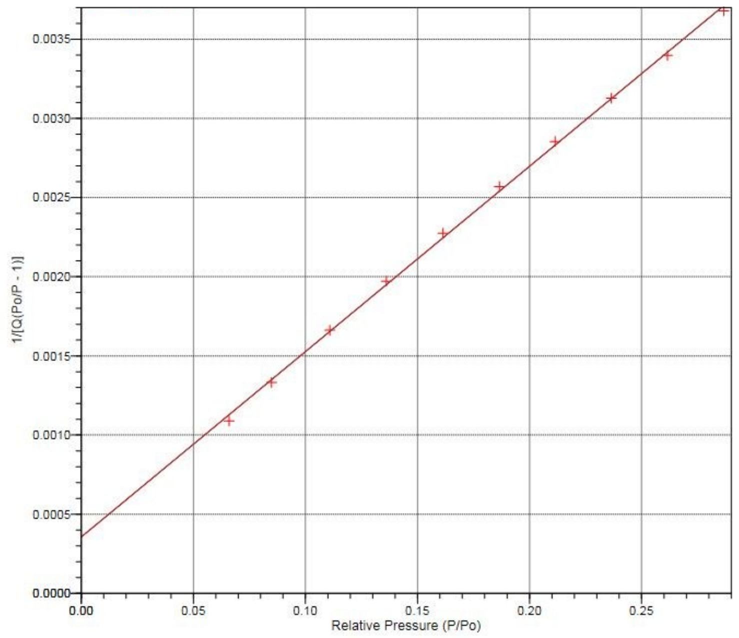

Figure S14. BET surface area of gold nanoclusters impregnated in mesoporous silica nanoparticles.

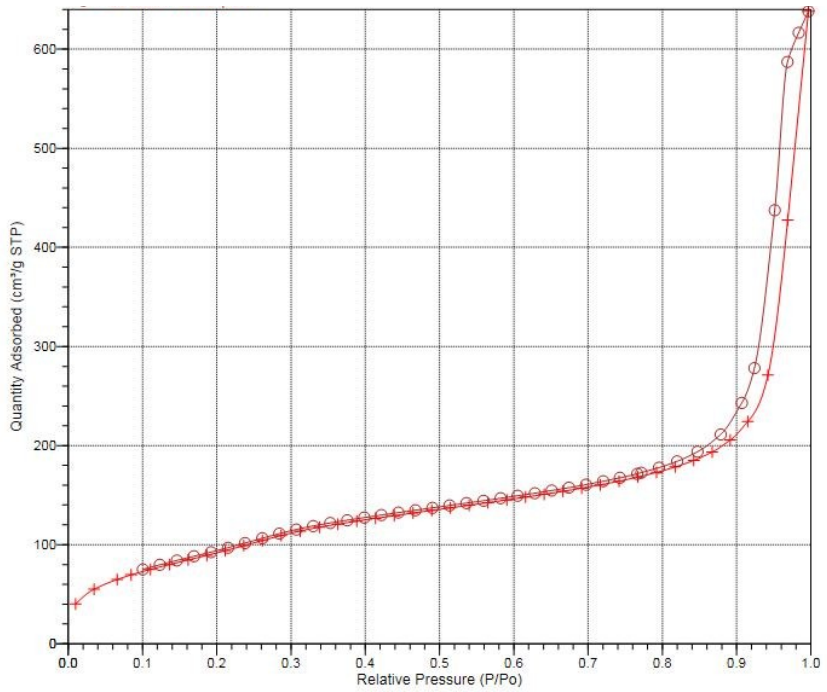

Figure S15. Isotherm linear plot of gold nanoclusters impregnated in mesoporous silica nanoparticles, showing type IV isotherm

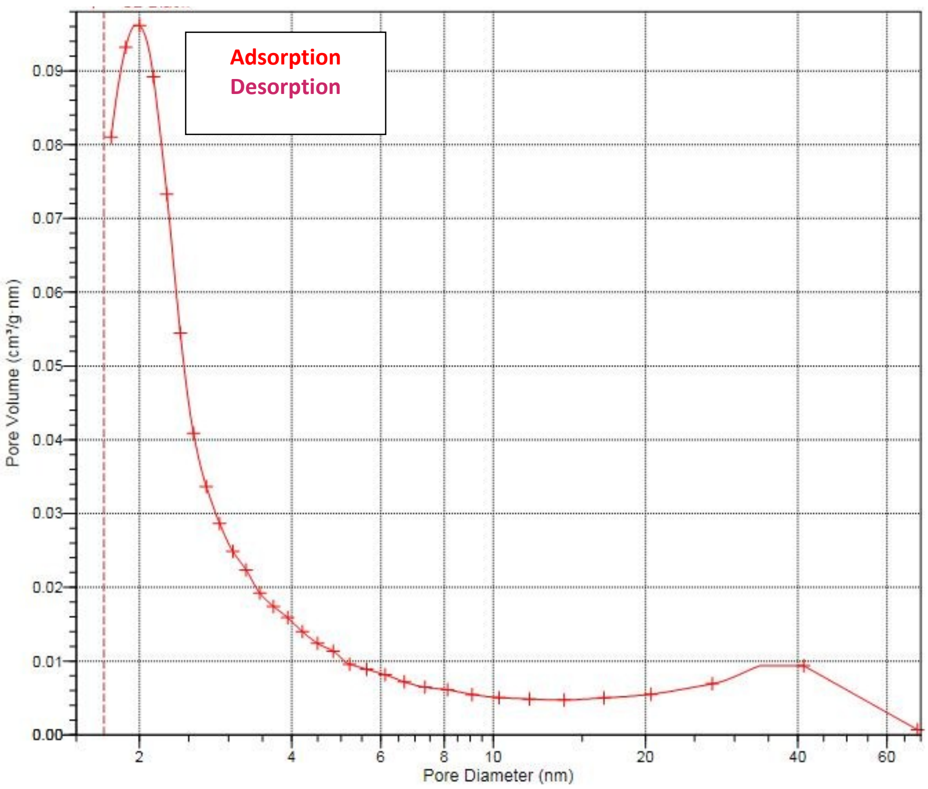

Figure S16. Differential pore volume of gold nanoclusters impregnated mesoporous silica nanoparticles determined using BJH model.

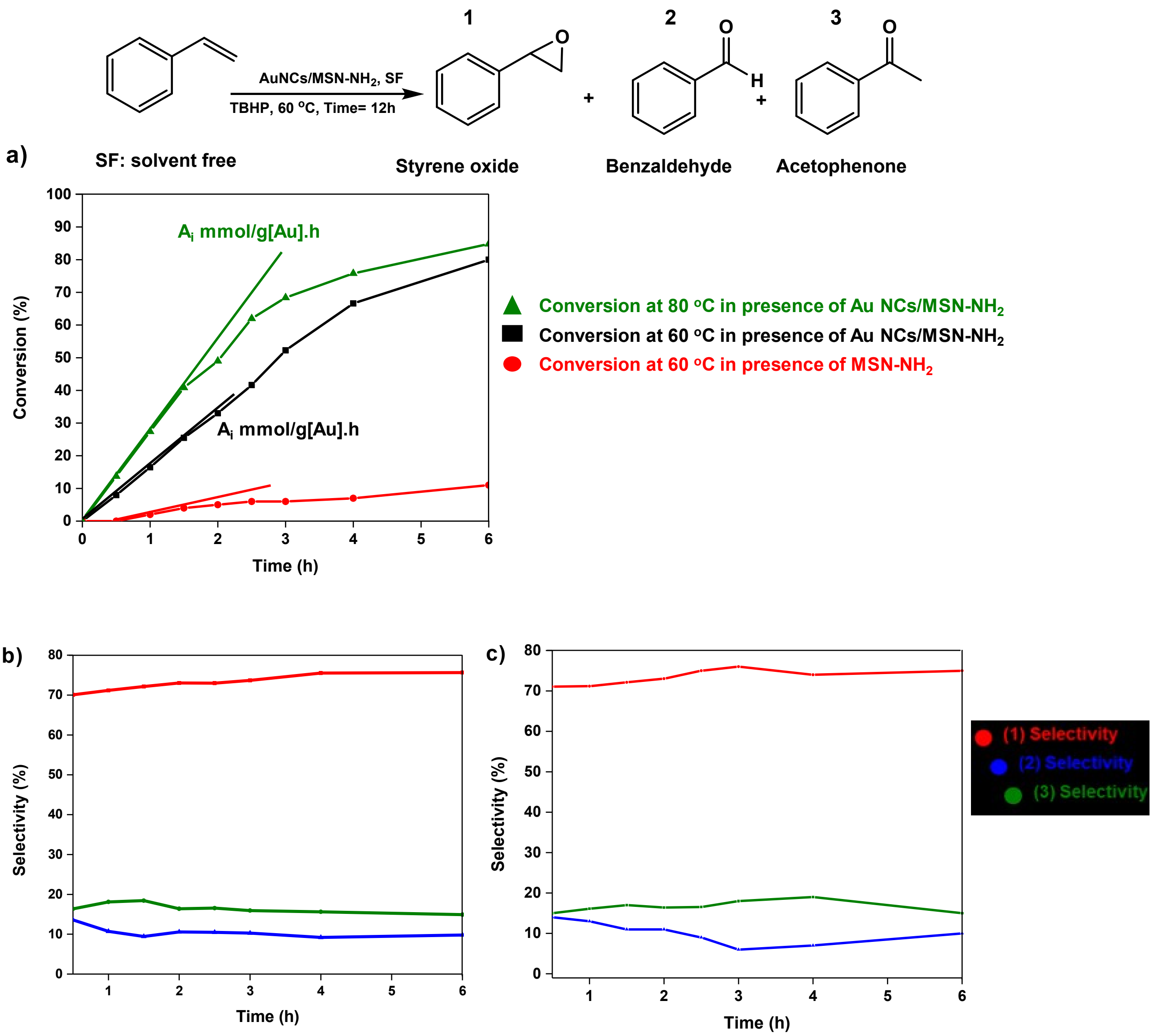

Figure S17. a) Kinetic study of the conversion of Styrene at 60 °C (black) and 80°C (green). **b)** The selectivity of Styrene epoxide (1), benzaldehyde (2) and acetophenone (3) at 80 °C during several hours**. c)** The selectivity of Styrene epoxide (1), benzaldehyde (2) and acetophenone (3) at 60 °C during several hrs**.**

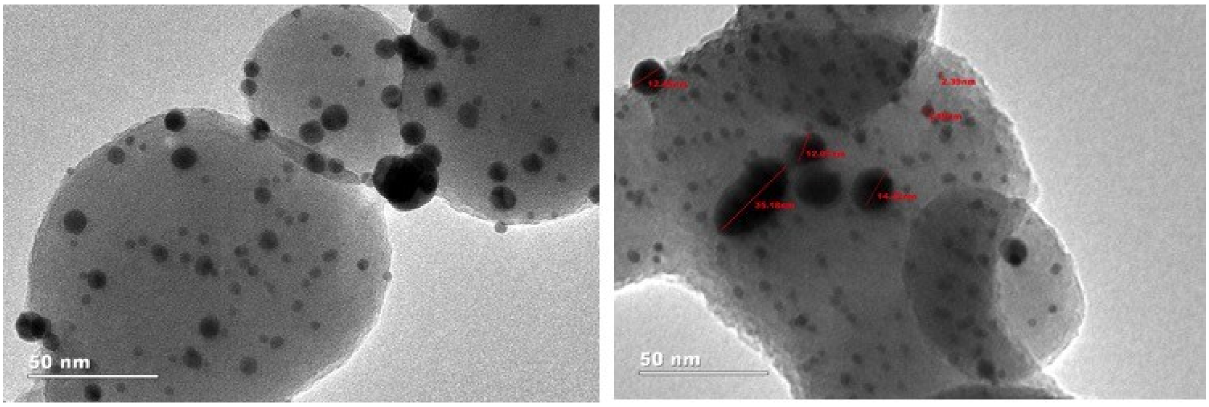

Figure S18. TEM images of AuNCs/MSN-NH_2 after using the catalyst for five runs. The small Au particles dissolve and redeposit on the surface of larger particles according to Ostwald ripening mechanism.

References

1. J. R. Kremer, D. N. Mastronarde and J. R. McIntosh, *Journal of structural biology*, 1996, **116**, 71-76.
2. K. K. Unger, *Porous silica*, Elsevier, 1979.
3. K. P. de Jong, *Synthesis of solid catalysts*, John Wiley & Sons, 2009.
